# Newly Diagnosed Acute Myeloid Leukemia in a Patient With Severe SARS-CoV-2 Infection

**DOI:** 10.7759/cureus.14480

**Published:** 2021-04-14

**Authors:** Panagiotis Papamichalis, Gerasimina Tsinti, Evangelia Papapostolou, Christos Hadjichristodoulou, Matthaios Speletas

**Affiliations:** 1 Intensive Care Unit, General Hospital of Larissa, Larissa, GRC; 2 Department of Immunology & Histocompatibility, Faculty of Medicine, School of Health Sciences, University of Thessaly, Larissa, GRC; 3 Department of Hygiene and Epidemiology, Faculty of Medicine, School of Health Sciences, University of Thessaly, Larissa, GRC

**Keywords:** covid-19, acute myeloid leukemia

## Abstract

We present a 68-year-old male patient with persistent and complicated SARS-CoV-2 infection who was diagnosed with acute myeloid leukemia (AML). The patient suffered from fever, cough and progressive dyspnea for 10 days and he was admitted to the intensive care unit due to respiratory failure and cytokine release syndrome (CRS). Despite a transient improvement of CRS by the implementation of supportive care, including also the administration of recombinant tissue plasminogen activator (rt-PA) and tocilizumab, his clinical course worsened over time. Thus, a bone marrow aspiration was performed revealing the presence of myeloblasts in a proportion of 32% and flow cytometry confirmed the diagnosis of AML-M1 according to FAB classification. Re-evaluation of peripheral blood tests revealed that the patient was admitted with anemia and thrombocytopenia that were never recovered during hospitalization. Due to the patient’s poor clinical condition, no chemotherapy was applied, and he died of sepsis and multi-organ failure two days later. This case suggests that in all patients with a persistent and/or complicated infection, even during pandemics, the presence of an underlying hematologic malignancy should always be taken into consideration.

## Introduction

The current SARS-CoV-2 pandemic (Coronavirus Disease 2019, COVID-19) is undoubtedly one of the greatest challenges in modern medicine; one for which the world was not prepared. Clinical and epidemiological data show that older patients with or without previous medical history, comprise the population group most vulnerable for the development of serious sequelae of the disease and poor prognosis [1,2]. There is increasing evidence that the human immune system slowly weakens with age (a phenomenon called immunosenescence) and, as a result, older individuals face difficulties in efficiently fighting off viral infections [3]. Additionally, individuals with a weak immune system, either from primary causes or secondary to other conditions, e.g. malignancies or immunosuppressive treatment for any etiology, are at risk of increased morbidity and fatality rates from common epidemics of viral etiology [4,5].

Recent reports described several patients with hematologic malignancies who also develop COVID-19 [6,7], but to the best of our knowledge, only two cases of COVID-19 with undiagnosed acute leukemia has been reported until now [8,9]. Such a similar case we describe below, suggesting that awareness for an underlying hematologic cancer should also be considered in cases of a persistent and complicated SARS-CoV-2 infection.

## Case presentation

**Figure 1:**
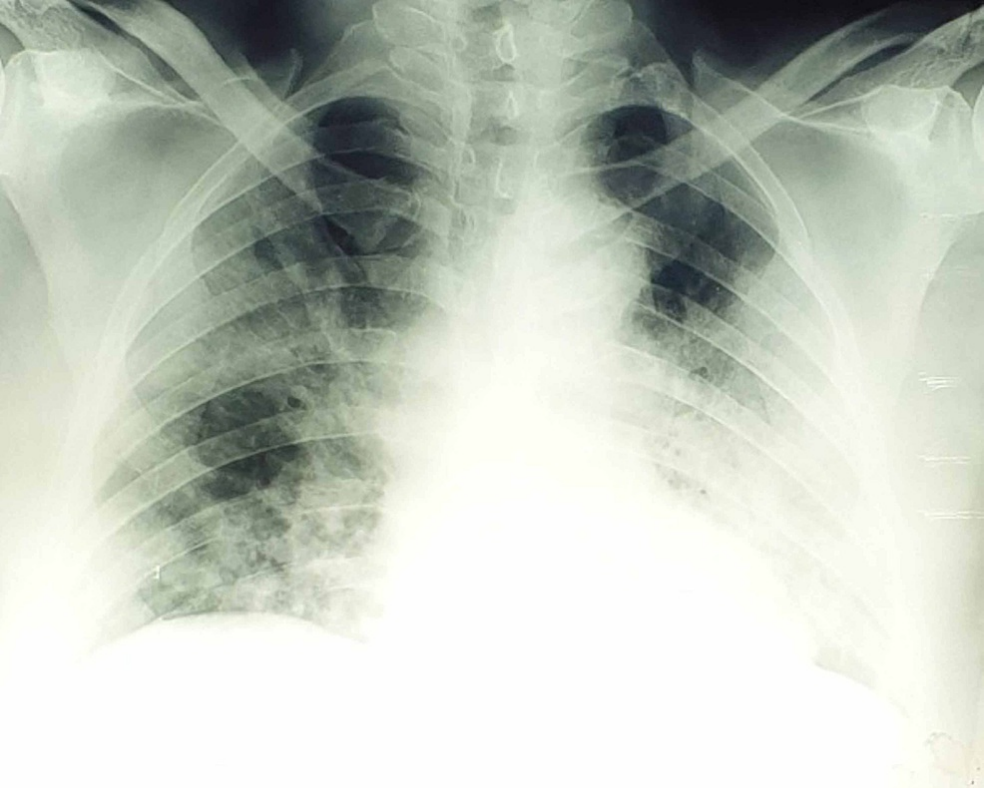
Chest radiograph of the patient with COVID-19 at diagnosis.

A 68-year-old male was admitted to the hospital due to fever, cough and progressive dyspnea for 10 days. The patient had received both antibiotic (clarithromycin) and anti-viral treatment (oseltamivir) by his family doctor, with no improvement. Even during the initial clinical evaluation, arterial blood gases revealed acute respiratory failure with multiple diffuse infiltrates in chest X-ray (Figure 1) and the patient was immediately admitted to the intensive care unit (ICU), with suspicion of severe acute respiratory syndrome coronavirus 2 (SARS-CoV-2) infection. The pharyngeal swab was taken, and real-time Polymerase Chain Reaction (rt-PCR) was positive for SARS-CoV-2, confirming the diagnosis of COVID-19. His family history was negative for COVID-19, suggesting that he was infected by social contact. Interestingly, the patient had only a medical history of mild hypertension, receiving tablets of candesartan-hydrochlorothiazide, and diabetes mellitus type 2, he was an ex-smoker (80 pys) and his lab exams one year ago were into normal limits.

**Figure 2:**
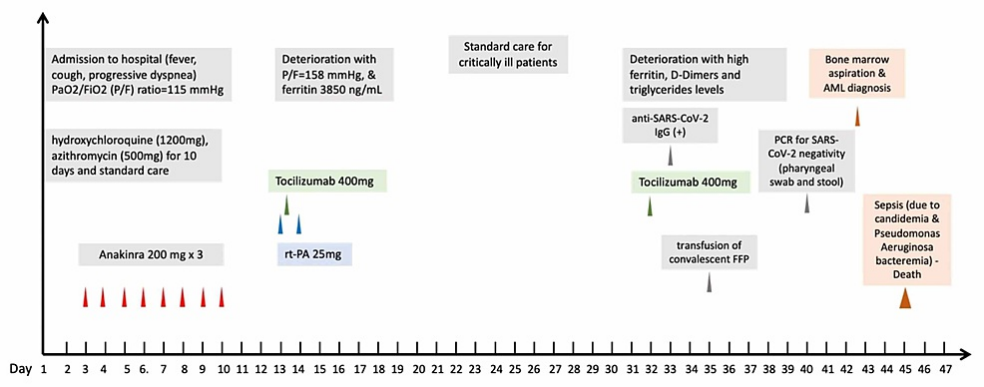
An overview of patient’s clinical course and management during hospitalization.

Laboratory tests at diagnosis (Table 1) suggested the presence of cytokine release syndrome (CRS) [10], while the patient’s clinical course, laboratory findings and outcome are presented in detail in a recent publication focused only on patient’s management with recombinant tissue plasminogen activator (rt-PA) and tocilizumab (an antagonist of interleukin-6 receptor) [11]. An overview of the patient’s clinical course and management is presented in Figure 2. It is worthy of note that during hospitalization, the patient also developed candidemia and bloodstream bacterial infections (by *Stenotrophomonas maltophilia* and *Pseudomonas aeruginosa*) that were encountered by broad-spectrum antifungal and antibiotic treatments.

**Table 1:** Laboratory findings of the patient at COVID-19 diagnosis Abbreviations: APTT, activated partial thromboplastin time; BUN, blood urea nitrogen; CPK, creatine phosphokinase; CRP, C reactive protein; INR, International Normalized Ratio; LDH, lactate dehydrogenase; PLT, platelets; SGOT, serum glutamate oxaloacetate transaminase; SGPT, serum glutamic pyruvic transaminase; WBC, white blood cell count. The values out of normal range are presented as “hi” or “low” accordingly. ^A peripheral blood smear was not evaluated at COVID-19 diagnosis *Normal range for males

Parameter	Value		Normal range
WBC (x 10^9^/L)	4.6		4.0-10.0
Neutrophils (x 10^9^/L)	1.5		1.5-7.0
Lymphocytes (x 10^9^/L)	1.0		1.0-4.0
Monocytes (x 10^9^/L)	2.0^	hi	0.3-0.9
Ht (%)	25.8		42.0-52.0*
Hb (gr/dL)	8.0		14.0-17.0*
PLT (x 10^9^/L)	40.0		140.0-400.0
Prothrombin time (sec)	13.4		11.0-13.5
INR	1.1		0.8-1.1
APTT (sec)	36.5		30.0-40.0
Fibrinogen (mg/dL)	500.0	hi	200.0-400.0
D-Dimers (ng/mL)	10.5	hi	<0.5
Glucose (mg/dL)	152		70.0-100.0
Creatinine	0.7		0.81-1.24
SGOT (U/L)	63.0	hi	5.0-40.0
SGPT (U/L)	53.0		7.0-56.0
LDH (U/L)	372.0	hi	140.0-280.0
CPK (U/L)	283.0		39-308 *
Albumin (g/dL)	3.3	low	3.5-5.5
Triglycerides (mg/dL)	169.0	hi	<150.0
Ferritin (ng/mL)	887.0	hi	20.0-250.0*
CRP (mg/L)	108.0	hi	<5.0
Potassium (mEq/L)	4.2		3.5-5.5
Sodium (mEq/L)	137.0		135.0-147.0
Calcium (mEq/L)	7.8	low	8.5-10.2

Three days after the last dosage of tocilizumab, the evaluation of the patient’s serum revealed the presence of anti-SARS-CoV-2 IgG antibodies (SARS-CoV-2 IgG assay, Abbott Laboratories Inc., Illinois, USA), as well as the negativity of PCR for SARS-CoV-2 in blood and stool. However, his clinical course worsened, while the lab tests revealed a sustained anemia (Hb: 8.2 gr/dL) and thrombocytopenia (platelet count: 24 x 109/L). At that time, a suspicion of subsequent bacterial infection and the emergence of the secondary hemophagocytic syndrome was suspected.

**Figure 3:**
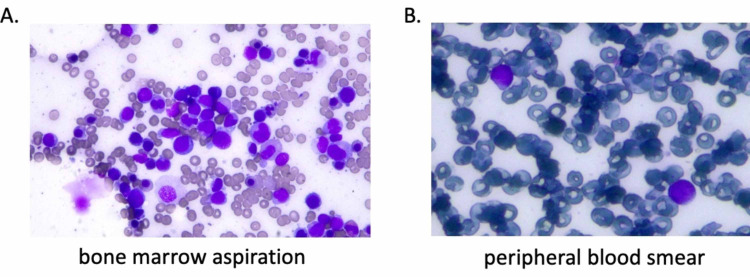
Patient with COVID-19 and a newly diagnosed acute myeloid leukemia. (A) Bone marrow aspiration smear. (B) Peripheral blood smear.

**Figure 4:**
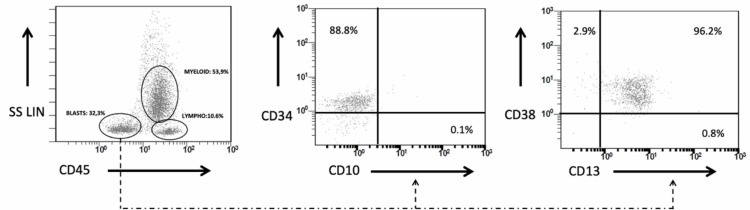
Flow cytometry immunophenotyping in the patient with COVID-19 and a newly diagnosed acute myeloid leukemia. The presence of myeloblasts is established in the plot of side scatter linear scale (SS LIN) and the cluster differentiation (CD)45 expression. Further analyses revealed the expression on myeloblasts of CD34, CD13 and CD38 markers.

Therefore, a bone marrow aspiration was performed and, surprisingly, the presence of myeloblasts in a proportion of 32% was observed (Figures 3 and 4), a finding that was confirmed in a new aspiration a day later. Immunophenotyping revealed that myeloblasts were positive for the expression of CD34, CD38, CD13, CD33, CD117 and HLA-DR molecules (Figure 4) and a diagnosis of acute myeloid leukemia (AML), M1 according to French-American-British (FAB) classification, was made. Re-evaluation of peripheral blood tests revealed that the patient was admitted with anemia and thrombocytopenia (Table 1) that were never recovered during hospitalization. Moreover, no peripheral blood (PB) evaluation had been performed until AML diagnosis, even though myeloblasts were also present there (Figure 3B). Due to the patient’s poor clinical condition, no chemotherapy was applied when AML diagnosed, and the patient died from sepsis and multi-organ failure two days later [11].

## Discussion

In this case study, we describe a patient with AML and SARS-CoV-2 infection. Bearing in mind that the patient’s medical history was negative for any hematologic disease, his progressive condition was initially attributed to COVID-19 and all medical efforts were focused on the management of the viral infection and its complications. In this context, no PB smear evaluation was performed after admission and the lab results were attributed only to COVID-19. Only when molecular analyses for SARS-CoV-2 in blood and stools became negative and anti-SARS-CoV-2 IgG antibodies were detected in the patient’s serum, a bone marrow aspiration was performed; nevertheless, even at that time, the initial assessment involved only infectious complications of SARS-CoV-2 infection. However, AML was diagnosed that clearly justifies the bad progression and outcome of the patient.

As described above, our patient received tocilizumab for the management of CRS, but he developed severe fungal and bacterial infections. It is worthy of note that the patient received the aforementioned medication in April and May 2020, while the recent guidelines for the management of COVID-19 patients suggest that the administration of tocilizumab should be avoided in patients with a platelet count below 50.000/mL (March 2021, https://www.covid19treatmentguidelines.nih.gov/statement-on-tocilizumab/). Moreover, recent studies have shown that although tocilizumab is effective in the management of CRS, reducing the mortality rate of COVID-19 patients, its administration is related to remarkable adverse events, including bacterial infections [12,13]. Thus, we could not exclude the possibility that the administration of tocilizumab may aggravate the immunosuppression of the underlying primary disease, resulting in the patient’s poor outcome.

As mentioned above, there are a few reports in the literature describing similar patients with undiagnosed acute leukemia and COVID-19 [8,9]. Although the diagnosis of leukemia, in the aforementioned studies, was performed earlier than our case, the prognosis of patients was also poor. This is in accordance with recent multicenter studies reporting that cancer patients have been affected by COVID-19 during the pandemic in a rather similar incidence to the general population; however, the outcome of SARS-CoV-2 infection is significantly worse in patients with hematologic malignancies [14,15].

## Conclusions

The patient presented herein, suffered from a persistent COVID-19 infection. However, we suggest that leukemia, perplexed by COVID-19, was the main causative of patient’s bad outcome. Our case further supports the notion, that in all cases of a persistent and/or complicated infection, even during pandemics, the presence of an underlying hematologic malignancy should always be taken into consideration.
